# Racial Discrimination and Metabolic Syndrome in Young Black Adults

**DOI:** 10.1001/jamanetworkopen.2024.5288

**Published:** 2024-04-01

**Authors:** Nia Heard-Garris, Tianyi Yu, Gene Brody, Edith Chen, Katherine B. Ehrlich, Gregory E. Miller

**Affiliations:** Department of Pediatrics, Northwestern University Feinberg School of Medicine, Chicago, Illinois; Division of Advanced General Pediatrics and Primary Care, Department of Pediatrics, Ann & Robert H. Lurie Children’s Hospital of Chicago, Chicago, Illinois; Mary Ann & J. Milburn Smith Child Health Research, Outreach, and Advocacy Center, Stanley Manne Children’s Research Institute, Ann & Robert H. Lurie Children’s Hospital of Chicago, Chicago, Illinois; Department of Psychology and Institute for Policy Research, Northwestern University, Evanston, Illinois; Center for Family Research, University of Georgia, Athens; Center for Family Research, University of Georgia, Athens; Department of Psychology and Institute for Policy Research, Northwestern University, Evanston, Illinois; Center for Family Research, University of Georgia, Athens; Department of Psychology, University of Georgia, Athens; Department of Psychology and Institute for Policy Research, Northwestern University, Evanston, Illinois

## Abstract

**IMPORTANCE:**

Metabolic syndrome (MetS) is a common health condition that predisposes individuals to cardiovascular disease (CVD) and disproportionately affects Black and other racially and ethnically minoritized people. Concurrently, Black individuals also report more exposure to racial discrimination compared with White individuals; however, the role of discrimination in the development of MetS over time and associated mediators in these pathways remain underexplored.

**OBJECTIVE:**

To evaluate the association between racial discrimination and MetS in rural Black individuals transitioning from late adolescence into early adulthood and to identify potential mediating pathways.

**DESIGN, SETTING, AND PARTICIPANTS:**

This longitudinal cohort study included Black adolescents enrolled in the Strong African American Families Healthy Adults (SHAPE) Project between June 2009 and May 2021. Families resided in rural counties of Georgia, where poverty rates are among the highest in the nation. Analyses included 322 of the 500 participants who originally enrolled in SHAPE and who were eligible to participate. Guardians provided information about socioeconomic disadvantage. Analyses were conducted in April 2023.

**EXPOSURES:**

Youths reported exposure to racial discrimination annually from ages 19 to 21 years.

**MAIN OUTCOMES AND MEASURES:**

MetS was the main health outcome and was measured at ages 25 and 31 years. MetS was diagnosed according to the International Diabetes Federation guidelines, which requires central adiposity (ie, waist circumference ≥94 cm for males and ≥80 cm for females) and at least 2 of the 4 additional components: signs of early hypertension (ie, systolic blood pressure ≥130 mm Hg or diastolic blood pressure ≥85 mm Hg); elevated triglyceride levels (ie, >150 mg/dL); elevated fasting glucose level (ie, ≥100 mg/dL); or lowered high-density lipoprotein levels (ie, <40 mg/dL in men and <50 mg/dL in women). At age 25 years, markers of inflammatory activity (ie, soluble urokinase plasminogen activator receptor [suPAR]) and sleep problems were collected to consider as potential mediators.

**RESULTS:**

In 322 participants (210 [65.2%] female) ages 19 to 21 years, more frequent exposure to racial discrimination was associated with higher suPAR levels (*b* = 0.006; 95% CI, 0.001-0.011; *P* = .01) and more sleep problems at age 25 years (*b* = 0.062; 95% CI, 0.028-0.097; *P* < .001) as well as a 9.5% higher risk of MetS diagnosis at age 31 years (odds ratio [OR], 1.10; 95% CI, 1.01-1.20; *P* = .03). Both suPAR (*b* = 0.015; 95% CI, 0.002-0.037) and sleep problems (*b* = 0.020; 95% CI, 0.002-0.047) at age 25 years were significant indirect pathways. No significant interactions between sex and discrimination emerged.

**CONCLUSIONS AND RELEVANCE:**

This study suggests that racial discrimination in late adolescence is associated with MetS among Black young adults through biobehavioral pathways. Thus, health interventions for MetS in Black adults will need to contend with sleep behaviors and inflammatory intermediaries as well as address and reduce exposure to racial discrimination to narrow disparities and promote health equity.

## Introduction

In the United States, 1 in 3 people have metabolic syndrome (MetS), with racially and ethnically minoritized groups having the highest prevalences.^1,2^ MetS comprises multiple adverse health factors,^3^ including central adiposity, hyperglycemia, dyslipidemia, and elevated blood pressure, that place individuals at higher risk for poor cardiometabolic health throughout their lifetime. Although these factors are distinct, they are interrelated and often found to co-occur, placing those affected at 5 times the risk of type 2 diabetes and twice the risk of developing cardiovascular disease within 5 to 10 years compared with peers without MetS.^3^ Initially MetS was thought to be secondary to rising levels of obesity and more sedentary lifestyles globally, then insulin resistance was later implicated.^3,4^ Briefly, abdominal fat cells increase free fatty acid levels in the body, creating a more proinflammatory state and neurohormonal activation, which may trigger insulin resistance; however, despite an acknowledgment of the lifestyle factors, particularly central adiposity related to overeating, as well as the additional genetic and epigenetic contributors, the exact pathogenesis of MetS is still debated.^4-6^

Although the mechanisms are still being elucidated, racial and ethnic disparities in the prevalence of MetS have been consistently documented.^1,2^ In nationally representative studies, non-Hispanic Black women were more likely than non-Hispanic White women to have MetS between 2007 and 2012, and between 2011 and 2016, Hispanic/Latinx people and multiracial groups had an increase in MetS prevalence.^1,2^ Furthermore, studies have shown that while MetS prevalence has been high, overall the prevalence has remained stable or even declined for multiple demographic groups, except for young adults.^1,5^ For young adults ages 20 to 39 years, MetS prevalence rates have increased,^1^ suggesting that the transition from adolescence to adulthood is an important developmental window to identify modifiable precursors of cardiometabolic dysregulation.

Racial discrimination has been proposed as one factor that may contribute to the development of MetS in Black and other racially and ethnically minoritized people.^7^ Discrimination occurs through both structural-level (ie, structural racism) and individual-level mechanisms and is often first experienced in childhood and adolescence.^8-10^ Black US adolescents especially are burdened, reporting over 5 experiences of racial discrimination daily via a variety of platforms.^11^ Emerging research suggests these earlier exposures to racial discrimination are associated with higher risks of cardiovascular disease in adulthood.^9^ However, there is a dearth of longitudinal analyses testing the association between earlier discrimination and subsequent MetS, and even less is known about behavioral and biological pathways that might underlie any such relationship. Filling these gaps in knowledge is an important step for health equity research because late adolescence and early adulthood are likely to be sensitive periods during which interventions could reduce the likelihood of MetS and subsequent type 2 diabetes and cardiometabolic diseases.

Therefore, in this article we sought to examine the prospective association between late adolescent racial discrimination and MetS in early adulthood. Specifically, this study leverages data from the Strong African Americans Health Adults Project (SHAPE), a longitudinal cohort study of African American individuals residing in rural counties in Georgia, beginning in fifth grade and followed until early adulthood, to examine how the racialized experiences of Black adolescents are associated with the development of MetS in early adulthood. Additionally, we explore 2 possible mechanisms that might help to explain how exposure to discrimination in adolescence forecasts MetS in young adulthood. Our analyses consider nonresolving inflammation, as indexed by soluble urokinase plasminogen activator receptor (suPAR), and sleep problems, both of which may predispose individuals to excess weight gain and cardiometabolic dysregulation that contribute to the subsequent onset of MetS.

## Methods

### Study Design

We used data from the SHAPE Project to conduct this longitudinal cohort study.^12^ Written informed consent was completed at all assessments.^12^ The University of Georgia’s institutional review board reviewed and approved all study procedures. This report follows the Strengthening the Reporting of Observational Studies in Epidemiology (STROBE) reporting guidelines for cohort studies.^13^

### Data and Study Population

SHAPE was originally designed as an intervention study that tested the effectiveness of the Strong African American Families (SAAF) program. This program focused on developing skills to enhance parenting, strengthen family relationships, and foster youth competencies via goal setting and resisting peer pressure in order to reduce substance use and risky behavior among Black adolescents. After the 7-week intervention, families were invited to participate in regular follow-up assessments, which have continued at regular intervals over the past 20 years. Target participants (now well into adulthood) have completed measures of psychosocial stress and support, and they have been providing blood samples and anthropometric assessments for physical health assessments. Starting in 2001, the SHAPE study enrolled 667 Black children in fifth grade (mean [SD] age, 11.2 [0.3] years; range, 11-13 years) along with their primary caregivers. Families resided in rural counties of Georgia where poverty rates were among the highest in the nation. At enrollment, primary caregivers had a median household income of $1612 per month, and 42.3% lived below federal poverty thresholds. Economically, the households of participants in this study can be characterized as working poor.^12^

In 2009 to 2010, when participants were aged 19 years, about 500 youths were randomly selected for a biomarkers substudy. In 2015 and 2021, when participants were aged 25 and 31 years, a Black field researcher visited their homes to administer a computerized survey and collect antecubital blood. The 322 participants who completed both assessments form the analytic sample of this study. Compared with the original cohort, the analytic sample had a higher percentage of female participants (210 of 322 [65.2%] vs 281 of 517 [54.4%]); the samples were similar on the other study variables (Table 1).

#### Discrimination

At ages 19, 20, and 21 years, participants reported their experiences with discrimination using the Schedule of Racist Events.^14^ This scale is used frequently with youths^15^ and contains 9 items assessing the frequency of exposure to events such as racial slurs, physical threats, and false accusations. Example items include, “Have you been ignored, overlooked, or not given service because of your race?”, “Have you been treated rudely or disrespectfully because of your race?”, and “Have you been watched or followed while in public because of your race?” Items were rated on a 0 (never happened) to 2 (happened a lot) scale and summed so that higher scores reflect more discrimination (Cronbach α, 0.91-0.92). Values were averaged from scores obtained at ages 19 to 21 years.

#### Sleep Problems

At age 25 years, participants completed the 6-item sleep problems scale from the Medical Outcome Study.^16^ Example items include, “Have trouble falling asleep,” “Awaken short of breath or with a headache,” and “Have trouble staying awake during the day.” Items were rated on a scale ranging from 1 (none of the time) to 6 (all of the time) and averaged such that higher scores indicated more sleep problems (Cronbach α, 0.64).

#### Low-Grade Inflammation

At age 25 years, a phlebotomist visited each participant’s home to perform an antecubital blood draw. To minimize circadian variation, venipuncture was performed between 8:00 am and 10:00 am. Participants fasted for 8 hours beforehand to minimize dietary influences. Blood was drawn into Serum Separator tubes (Becton-Dickinson). Specimens were centrifuged on site at 1500 g for 20 minutes. The serum was harvested, divided into aliquots, frozen on dry ice, and driven back to the lab, where it was stored at −80 °C. At the end of the study, technicians measured soluble urinokinase plasminogen activator receptor (suPAR) in duplicate by commercial immunoassay (Human Quantikine ELISA). The lower limit of detection was 33 pg/mL. Across runs, the mean intra-assay coefficient of variation for duplicate pairs was 1.5%, and the interassay coefficients of variation was 1.1%. When a sample value was greater than the highest standard, we diluted and reassayed. suPAR was skewed, so we normalized its distribution using a log-10 transformation before analysis.

We considered including other inflammatory biomarkers in this analysis, such as C-reactive protein, interleukin-6, or tumor necrosis factor-α. However, as we reported in an earlier analysis of this sample,^17^ suPAR was the only inflammatory biomarker that showed a consistent prospective association with late-adolescent racial discrimination. Moreover, mounting evidence from studies in Black populations suggests that suPAR is a stronger predictor of morbidity and mortality from cardiometabolic disease than other inflammatory biomarkers.^18,19^ Given these observations, and the desire to minimize the risk of false discoveries related to multiple testing, we limited analyses of inflammation to suPAR.

#### MetS

At ages 25 and 31 years, signs of MetS were assessed at home visits. Fasting antecubital blood was used to measure glucose, triglyceride, and high-density lipoprotein levels on a Roche/Hitachi cobas c502 analyzer (age 25 years) and a Beckman Coulter AU5800 analyzer (age 31 years). Resting blood pressure was monitored while the participant sat reading quietly with a Critikon Dinamap Pro 100. Three readings were taken every 2 minutes, and the average of the last 2 readings was used as the resting index. The field researcher measured waist circumference twice at the midpoint of the upper iliac crest and lower costal margin, at the midaxillary line. If readings differed by 1 cm, they were repeated, and the closest 2 values were averaged.

MetS was diagnosed according to the International Diabetes Federation guidelines.^20^ These criteria specify that in adults, a MetS diagnosis requires central adiposity, which for the Black participants in this sample is defined as a waist circumference of 94 cm or greater for males and 80 cm or greater for females. At least 2 of the 4 additional components must also be present. They include (1) signs of early hypertension (systolic blood pressure ≥130 mm Hg or diastolic blood pressure ≥85 mm Hg), (2) elevated triglyceride level (>150 mg/dL [to convert to millimoles per liter, multiply by 0.0113]), (3) elevated fasting glucose level (≥100 mg/dL [to convert to millimoles per liter, multiply by 0.0555]), or (4) lowered high-density lipoprotein levels (<40 mg/dL in men and <50 mg/dL in women [to convert to millimoles per liter, multiply by 0.0259]).

#### Covariates

A panel of covariates was included in analyses to minimize confounding risks. The covariates include sex, coded as 1 if male and 0 if female, and family socioeconomic (SES) disadvantage from age 19 to 21 years, defined by a composite of 6 indicators including family poverty, primary caregiver’s noncompletion of high school or an equivalent, primary caregiver current unemployment, single-parent family structure, current receipt of Temporary Assistance for Needy Families, and income rated by the caregiver as inadequate to meet all needs, all coded 0 if absent and 1 if present, such that higher numbers indicate greater SES disadvantage.^12^ The SHAPE cohort was initially recruited to participate in a randomized clinical trial of a family-oriented intervention to prevent youth behavior problems and substance abuse. Participation in the intervention was not associated with any of the study outcomes. To minimize any residual confounding, however, we included a dichotomous covariate reflecting intervention condition (treatment vs control) in all models.

### Statistical Analysis

Data analysis was completed in April 2023. We used linear regression models for suPAR and sleep outcomes and logistic regression models for MetS outcomes to examine the association between racial discrimination and MetS. In adjusted analyses, we controlled for the panel of covariates described previously. Significance tests were 2-tailed, with α = .05. *P* values were 2-sided, and statistical significance was set at .05.

The mediational hypotheses involving suPAR and sleep were evaluated using a regression-based procedure.^16^ Briefly, regression coefficients were calculated for the associations of racial discrimination with suPAR and sleep problems (path A) and for the associations of suPAR and sleep problems with MetS diagnosis (path B). The indirect effect in which suPAR and sleep problems serve as the mediators connecting racial discrimination to MetS diagnosis was quantified as the product of the 2 regression coefficients (A × B). In addition, nonparametric bootstrapping was used to obtain the bias-corrected and accelerated confidence intervals of parameter estimates for significance testing.^21^ The parameter estimate was calculated 5000 times using random sampling with replacement to build a sampling distribution. All analyses were conducted using SPSS version 28 (IBM Corp) and the statistical macro package PROCESS.^22^

## Results

Table 1 displays characteristics of the analytic sample and compares it with the subset of participants who were excluded from this analysis because of missing data (195 [37.7%]). Our analytic sample included 210 female participants (65.2%), and 193 (59.9%) were intervention participants; the socioeconomic disadvantage mean (SD) was 2.82 (1.50), and racial discrimination mean (SD) score was 2.92 (2.90). The samples had similar distributions of the principal variables considered here, apart from sex, in that female participants were more likely to complete relevant assessments. Compared with participants with missing data, there were no statistically significant differences in intervention participation, socioeconomic disadvantage, or racial discrimination. The prevalence of MetS in the sample at age 25 years was 18.6% (60 participants). By age 31 years, this figure had roughly doubled to 36.6% (118 participants).

### Unadjusted Analysis

For descriptive purposes, Table 2 shows bivariate associations among principal variables. Male participants had lower suPAR and fewer MetS diagnoses than female participants. Socioeconomic disadvantage was associated with less racial discrimination between ages 19 and 21 years but presaged more sleep problems and higher MetS rates at age 25 years. As expected, racial discrimination between ages 19 and 21 years was associated with more suPAR and sleep problems at age 25 years (correlation coefficient, 0.116 at age 25 years), and these variables were, in turn, associated with higher likelihood of MetS at age 31 years (suPAR at age 25 years: correlation coefficient, 0.233; sleep problems at age 25 years: correlation coefficient, 0.158).

### Adjusted Analyses

Table 3 displays the results of regression models considering whether exposure to racial discrimination from ages 19 to 21 years is associated with suPAR levels and sleep problems at age 25 years and MetS at age 31 years. These models were adjusted for sex, participation in the SAAF intervention, and family socioeconomic disadvantage. The model for MetS diagnosis at age 31 years was also adjusted for MetS diagnosis at age 25 years, when the hypothesized mediators were assessed. As the coefficients illustrate, more frequent exposure to racial discrimination was associated with higher subsequent suPAR (*b* = 0.006; 95% CI, 0.001-0.011) and more sleep problems at age 25 years (*b* = 0.062; 95% CI, 0.028-0.097) as well as greater likelihood of MetS diagnosis at age 31 years (*b* = 0.095; 95% CI, 0.011-0.179). The coefficients help contextualize the magnitude of the associations: for every 1-SD increase in racial discrimination reported from ages 19 to 21 years, there was a 0.14-SD increase in suPAR levels and 0.20-SD increase in sleep problems at 25 years, and a 9.5% higher risk of MetS diagnosis at 31 years.

We used Hayes PROCESS macro, which is a regression-based approach for testing the plausibility of mediational scenarios to determine whether racial discrimination forecasts subsequent MetS through sleep problems and/or inflammatory activity.^22^ The Figure depicts the results of these analyses. As hypothesized, there were significant indirect pathways connecting more frequent racial discrimination with a greater likelihood of subsequent MetS through higher levels of both suPAR and sleep problems at age 25. These 2 indirect pathways were statistically distinct and similar in magnitude, as reflected in their coefficients (pathway through suPAR: coefficient, 0.0147; 95% CI, 0.0019-0.0367; pathway through sleep: coefficient, 0.0195; 95% CI, 0.0017-0.0466).

Because some previous analyses have suggested that discrimination may have a stronger association with health in males than females,^17^ we reestimated these models with an additional term representing the effect moderated by sex. However, we did not observe any significant sex × discrimination interactions in models projecting suPAR (*b* = −0.002; *P* = .76); sleep problems (b = −0.016; *P* = .66), or MetS diagnosis (*b* = −0.011; *P* = .90). There also were no significant 3-way interactions between sex, discrimination, and the presumptive mediators for MetS (suPAR × sex × discrimination: *b* = −0.039; *P* = .99; sleep problems × sex × discrimination: *b* = 0.198; *P* = .50).

## Discussion

In this prospective analysis of rural Black youths, we found a positive association between experiencing racial discrimination in late adolescence and MetS at age 31 years. Furthermore, this association was mediated by higher inflammation (suPAR) and sleep problems at age 25 years. Previous studies have found an association between discrimination and MetS in adulthood.^23,24^ Notably, our study adds to this literature in 2 meaningful ways. First, we estimated these associations using a prospectively designed and conducted study in which racial discrimination was measured annually for 3 years in late adolescence and associated with MetS over 6 and 12 years later. Second, our analyses considered mechanisms that might help to explain how racial discrimination contributes to MetS onset. The results suggest that sleep problems and inflammatory processes may serve as independent processes through which exposure to discrimination forecasts later cardiometabolic dysregulation.

Discrimination has been shown to increase cognitive, emotional, and physiological arousal, all of which can contribute to poor sleep.^25-28^ Over time, these sleep disturbances may have adverse effects on metabolism, which could serve as a clear pathway through which poor sleep contributes to MetS.^29^ Similarly, research has revealed that discrimination is linked to markers of inflammation, and inflammation’s role in the pathogenesis of cardiometabolic diseases is well understood.^9,17^ If these mediational findings are substantiated in future research, sleep problems and mechanisms to reduce inflammation could be explored as targets for preventive interventions aimed at reducing the disproportionate burden of MetS in Black US residents. These interventions would need to be developed in conjunction with larger-scale changes in policies that unjustly discriminate against and uniquely disadvantage Black US residents. However, while those policy changes are being pursued, interventions that target modifiable biobehavioral processes—like sleep difficulties and inflammation—could accelerate efforts to achieve health equity.

This association between racial discrimination and MetS may at least partly explain the racial and ethnic disparities seen in MetS. Trends of MetS indicate that while nearly 33% of US adults meet the criteria for it, there has been an increased prevalence for non-Hispanic Black people, which is not explained by the increased rates of obesity.^2^ Concerningly, however, primary public health prevention strategies have focused on improving fruit and vegetable access, encouraging physical activity, and health care access^2^ and have not emphasized the risk associated with living in toxic social environments. Our findings suggest that addressing nutritional needs, sedentary lifestyles, and lack of health care may not be adequate to reduce or prevent MetS in racial or ethnic minoritized populations. Specifically, interventions that do not measure or address the racial discrimination individuals experience may be ineffective.

### Limitations

This study must be interpreted within the context of its limitations. First, this sample focused on early adult Black participants in the rural South, and the unique sociocultural contexts in which they are living may have shaped the associations observed in this study. Thus, this study does not account for other racially and ethnically minoritized populations who may experience racial discrimination. Additionally, we had greater attrition of male participants in the study, which could have contributed to a biased analytic sample. Furthermore, we relied on self-reports of sleep problems, which often only weakly correlate with actigraphy and other behavioral measures of sleep.^30^ Future research should incorporate multiple methods to measure sleep to identify which aspects of sleep health are particularly affected by discrimination (eg, sleep onset, number of wakings per night, short sleep duration). Additionally, we only examined suPAR, given our previous finding demonstrating an association between suPAR and racial discrimination in this sample.^17^ However, there may be other inflammatory biomarkers that mediate the pathway between racial discrimination and MetS and require further exploration. Furthermore, we did not find an interaction between sex and discrimination; although in our previous work, we found that African American males with high levels of discrimination had increased suPAR trajectories over time and this association was not present in females, signifying that this finding needs to be further explored.

## Conclusions

In this study, Black individuals who experienced racial discrimination during late adolescence had an increased risk of MetS in adulthood, which may have been mediated by inflammation and sleep difficulties. These associations suggest that racial discrimination has persistent and detrimental effects on health. These findings also generate additional questions about the association of racial discrimination with MetS in other racial and ethnic groups in the United States. Future research should further examine these associations and interrogate opportunities for intervention, using sleep as a potential lever for prevention. Strategies to prevent and reduce racial discrimination exposure and associated sequelae are critical for improving health among racially and ethnically minoritized populations and moving us closer toward health equity.

## Supplementary Material

statement

## Figures and Tables

**Figure.**
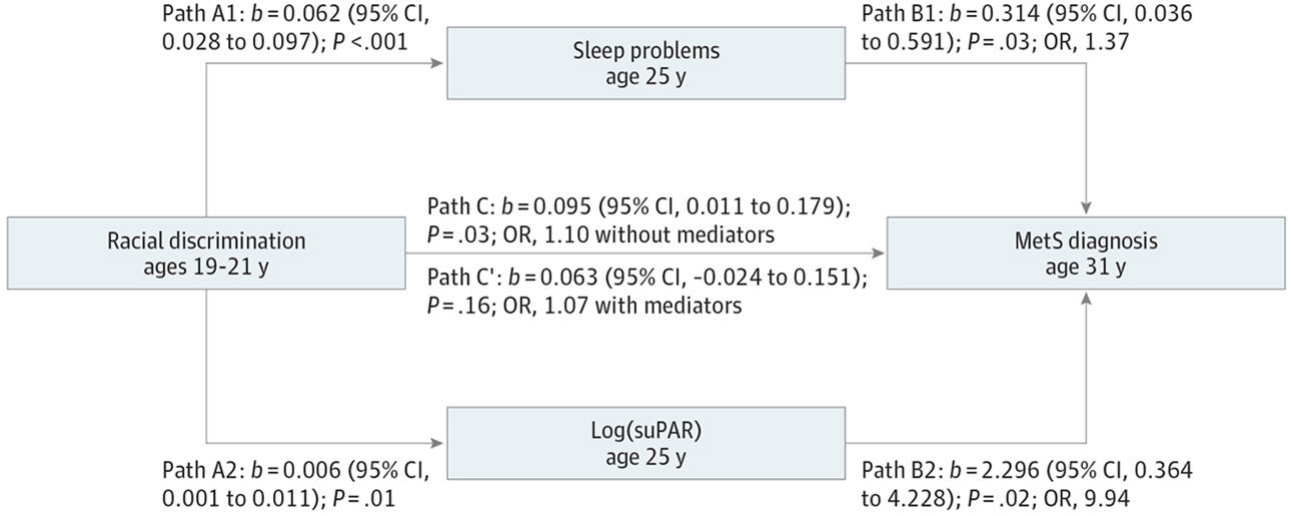
Sleep Problems and Soluble Urinokinase Plasminogen Activator Receptor (suPAR) at Age 125 Years as the Mediators of the Association Between Racial Discrimination at Ages 19 to 21 Years and Metabolic Syndrome (MetS) Diagnosis at Age 31 Years Model is adjusted for MetS at age 25 years, socioeconomic disadvantage at ages 19 to 21 years, sex, and receipt of Strong African American Families intervention status. Unstandardized coefficients (*b*) with 95% CIs are presented. A total of 322 participants were included.

**Table 1.** Comparison of Analytic Sample With Sample Excluded Due to Missing Data

Characteristic	Participants, No. (%)		χ^2^	*P* value
	Analytic sample	Cases missing data		
**Age 19-21 y (n = 517)**				
No.	322	195	NA	NA
Sex				
Male	112 (34.8)	124 (63.6)	40.62	<.001
Female	210 (65.2)	71 (36.4)	40.62	<.001
SAAF participant	193 (59.9)	106 (54.4)	1.55	.23
Socioeconomic disadvantage, mean (SD) (range 1-6)	2.84 (1.50)	2.77 (1.61)	*t* = 0.50	.61
Racial discrimination, mean (SD) (range 0-18)	2.92 (2.90)	3.26 (3.27)	*t* = −1.30	.20
**Age 25 years (n = 391)**				
No.	322	69	NA	NA
Log suPAR, mean (SD), pg/mL	3.33 (0.13)	3.32 (0.11)	*t* = −0.60	.55
Sleep problems, mean (SD), range 1-6	2.49 (0.91)	2.62 (0.89)	*t* = −1.23	.22
Metabolic syndrome diagnosis	60 (18.6)	7 (10.1)	2.88	.11
**Age 31 years (n = 344)**				
No.	322	22	NA	NA
Metabolic syndrome diagnosis	118 (36.6)	9 (40.9)	0.16	.82

Abbreviations: NA, not applicable; SAAF, Strong African American Families intervention; suPAR, soluble urokinase plasminogen activator.

**Table 2.** Correlations Among Study Variables Among 322 Participants

Variable	Correlation coefficient						
	Male sex	SAAF participant	Mean socioeconomic disadvantage	Mean racial discrimination	Log suPAR	Sleep problems	MetS, age 25 y
Male sex	NA	NA	NA	NA	NA	NA	NA
SAAF participant	−0.042	NA	NA	NA	NA	NA	NA
Mean socioeconomic disadvantage, ages 19-21 y	−0.022	0.159^a^	NA	NA	NA	NA	NA
Mean racial discrimination, ages 19-21 y	0.024	−0.064	−0.194^b^	NA	NA	NA	NA
Log suPAR, age 25 y	−0.238^b^	−0.017	0.103	0.116^c^	NA	NA	NA
Sleep problems, age 25 y	−0.059	0.029	0.115^c^	0.170^a^	0.006	NA	NA
MetS diagnosis, age 25 y	−0.115^c^	0.049	0.118^a^	−0.003	0.216^b^	0.072	NA
MetS diagnosis, age 31 y	−0.136^c^	−0.049	0.055	0.093	0.233^b^	0.158^a^	0.282^b^

Abbreviations: MetS, metabolic syndrome; NA, not applicable; SAAF, Strong African American Families program; suPAR, soluble urokinase plasminogen activator.

a *P* < .01.

b *P* < .001.

c *P* < .05.

**Table 3.** Association of Covariates and Discrimination With Subsequent suPAR, Sleep Problems, and MetS Diagnosis Among 322 Participants

Covariate	Log suPAR, age 25 y		Sleep problems, age 25 y		MetS diagnosis, age 31 y	
	*b* (95% CI)	β	*b* (95% CI)	β	*b* (95% CI)	OR (95% CI)
Male sex	−0.053 (−0.083 to −0.023)^a^	−0.187	−0.096 (−0.302 to 0.110)	−0.050	−0.541 (−1.06 to −0.020)^b^	0.58 (0.35 to 0.98)
SAAF participant	−0.014 (−0.044 to 0.015)	−0.051	0.033 (−0.168 to 0.235)	0.018	−0.338 (−0.834 to 0.158)	0.71 (0.43 to 1.17)
Socioeconomic disadvantage age 19-21 y	0.010 (0.001 to 0.020)^b^	0.116	0.087 (0.019 to 0.155)^b^	0.143	0.087 (−0.079 to 0.254)	1.09 (0.92 to 1.29)
MetS diagnosis ae 25 y	0.049 (0.012 to 0.086)^c^	0.142	0.116 (−0.138 to 0.371)	0.050	1.407 (0.798 to 2.017)^c^	4.08 (2.22 to 7.52)
Racial discrimination age 19-21 y	0.006 (0.001 to 0.011)^b^	0.137	0.062 (0.028 to 0.097)^c^	0.198	0.095 (0.011 to 0.179)^b^	1.10 (1.01 to 1.20)

Abbreviations: MetS, metabolic syndrome; OR, odds ratio; SAAF, Strong African American Families program; suPAR, soluble urokinase plasminogen activator.

a *P* < .001.

b *P* < .05.

c *P* < .01.

## Data Availability

See the Supplement.

## Key Points

### Question

Is racial discrimination associated with metabolic syndrome (MetS) in Black individuals during young adulthood, and what mechanisms connect them?

### Findings

In this cohort study with 322 participants, there was an association between racial discrimination in late adolescence and MetS in early adulthood, and path analyses were consistent with inflammatory activity and sleeping difficulties mediating this association.

### Meaning

These findings suggest that racial discrimination predisposes Black young adults to MetS via sleep problems and inflammation, which may serve as actionable targets for prevention in minoritized populations that could reduce existing disparities and promote health equity.

## Abbreviations

NA: not applicable
SAAF: Strong African American Families intervention
suPAR: soluble urokinase plasminogen activator
