# Supplementary material for: Racial Discrimination and Metabolic Syndrome in Young Black Adults
Source: JAMA Netw Open. Author manuscript; Available in PMC 2024 Jul 8. (PMC11229094; doi:10.1001/jamanetworkopen.2024.5288)
Supplement: statement [file NIHMS1999872-supplement-statement.pdf]

Data Sharing Statement

Heard-Garris. Racial Discrimination and Metabolic Syndrome in Young Black Adults. *JAMA Netw Open*. Published April 15, 2024. doi:10.1001/jamanetworkopen.2024.5288

Data
Data available: Yes
Data types: Deidentified participant data
How to access data: Data will be available upon request from the Principal Investigator, Katherine B Ehrlich (kehrlich@uga.edu).
When available: With publication

Supporting Documents
Document types: None

Additional Information
Who can access the data: Researchers whose proposed use of the data has been approved.
Types of analyses: Research and educational purposes only.
Mechanisms of data availability: Data will be made available after approval of a proposal, with investigator support, and signed data access agreement.
Any additional restrictions: NA
